# Deciphering the microbial landscape of lower respiratory tract infections: insights from metagenomics and machine learning

**DOI:** 10.3389/fcimb.2024.1385562

**Published:** 2024-05-23

**Authors:** Jiahuan Li, Anying Xiong, Junyi Wang, Xue Wu, Lingling Bai, Lei Zhang, Xiang He, Guoping Li

**Affiliations:** ^1^ Clinical Medicine Department, North Sichuan Medical College, Nanchong, China; ^2^ Laboratory of Allergy and Precision Medicine, Chengdu Institute of Respiratory Health, the Third People’s Hospital of Chengdu, Affiliated Hospital of Southwest Jiaotong University, Chengdu, China; ^3^ Department of Pulmonary and Critical Care Medicine, Chengdu third people’s hospital branch of National Clinical Research Center for Respiratory Disease, Affiliated Hospital of ChongQing Medical University, Chengdu, China; ^4^ National Center for Respiratory Medicine, National Clinical Research Center for Respiratory Disease, State Key Laboratory of Respiratory Disease, Guangzhou Institute of Respiratory Health, The First Affiliated Hospital of Guangzhou Medical University, Guangzhou, China

**Keywords:** metagenome, lower respiratory tract infection, respiratory microorganisms, machine learning, feature engineering

## Abstract

**Background:**

Lower respiratory tract infections represent prevalent ailments. Nonetheless, current comprehension of the microbial ecosystems within the lower respiratory tract remains incomplete and necessitates further comprehensive assessment. Leveraging the advancements in metagenomic next-generation sequencing (mNGS) technology alongside the emergence of machine learning, it is now viable to compare the attributes of lower respiratory tract microbial communities among patients across diverse age groups, diseases, and infection types.

**Method:**

We collected bronchoalveolar lavage fluid samples from 138 patients diagnosed with lower respiratory tract infections and conducted mNGS to characterize the lung microbiota. Employing various machine learning algorithms, we investigated the correlation of key bacteria in patients with concurrent bronchiectasis and developed a predictive model for hospitalization duration based on these identified key bacteria.

**Result:**

We observed variations in microbial communities across different age groups, diseases, and infection types. In the elderly group, *Pseudomonas aeruginosa* exhibited the highest relative abundance, followed by *Corynebacterium striatum* and *Acinetobacter baumannii*. *Methylobacterium* and *Prevotella* emerged as the dominant genera at the genus level in the younger group, while *Mycobacterium tuberculosis* and *Haemophilus influenzae* were prevalent species. Within the bronchiectasis group, dominant bacteria included *Pseudomonas aeruginosa*, *Haemophilus influenzae*, and *Klebsiella pneumoniae*. Significant differences in the presence of *Pseudomonas phage JBD93* were noted between the bronchiectasis group and the control group. In the group with concomitant fungal infections, the most abundant genera were *Acinetobacter* and *Pseudomonas*, with *Acinetobacter baumannii* and *Pseudomonas aeruginosa* as the predominant species. Notable differences were observed in the presence of *Human gammaherpesvirus 4*, *Human betaherpesvirus 5*, *Candida albicans*, *Aspergillus oryzae*, and *Aspergillus fumigatus* between the group with concomitant fungal infections and the bacterial group. Machine learning algorithms were utilized to select bacteria and clinical indicators associated with hospitalization duration, confirming the excellent performance of bacteria in predicting hospitalization time.

**Conclusion:**

Our study provided a comprehensive description of the microbial characteristics among patients with lower respiratory tract infections, offering insights from various perspectives. Additionally, we investigated the advanced predictive capability of microbial community features in determining the hospitalization duration of these patients.

## 1 Introduction

Lower respiratory tract infections are prevalent worldwide, encompassing a spectrum of severity from acute bronchitis to severe pneumonia (Mizgerd, 2006, 2008). However, these infections can be attributed to single or multiple microorganisms, exhibiting a range of virulence from commensal to highly pathogenic (De Roux, 2006; Webster and Govorkova, 2006; Weber et al., 2007). Accurate identification of the causative microorganisms is imperative for effective treatment and prevention of complications. The rapid advancement of mNGS technology offers a more sensitive detection method for pathogenic microorganisms compared to traditional microbiological techniques (Miao et al., 2018). The lung microbiota plays a vital role in maintaining respiratory health and influencing the severity of lower respiratory tract diseases (Fenn et al., 2022). Although studies have explored the characteristics of lung microbiota across different severity levels of lung infections using metagenomics (Zhan et al., 2023), multidimensional analysis of lung microbiota characteristics remains limited.

Machine learning algorithms are algorithms designed to automatically analyze data, uncover patterns, and predict unknown data based on these patterns. They exhibit robust fitting and generalization capabilities, particularly for classification tasks involving complex features. Integration of machine learning into medicine holds the promise of delivering more accurate diagnoses and personalized treatments for patients (Weiss et al., 2012, 2015). By combining machine learning with mNGS, our objective is to address practical clinical challenges from a microbial standpoint. This involves comprehensively elucidating patient microbiota characteristics and leveraging machine learning predictive models.

In this study, to explore the characteristics of patients with lower respiratory tract infections, we grouped the patient with different criteria, such as age and comorbid conditions. We analyzed the microbial differences among various groups based on different classification criteria and investigated the correlations and predictive capabilities of the microbiota using machine learning, specifically focusing on the prediction of hospitalization duration.

## 2 Methods

### 2.1 Study population

This study enrolled 157 patients with lower respiratory tract infections treated at the Respiratory and Critical Care Medicine Department of Chengdu Third People’s Hospital from March 1 to June 30, 2023. Following a thorough evaluation by two seasoned clinicians, 138 patients were selected for inclusion. The research methodology entailed prospective specimen collection and subsequent blinded retrospective analysis, adhering to the principles of the Helsinki Declaration. Participants provided written informed consent, and the study’s protocols received approval from the Chengdu Third People’s Hospital Institutional Review Board, ensuring compliance with all pertinent ethical standards. The characterization of lower respiratory tract infections is based on the criteria outlined in Huang et al. (Huang et al., 2018).

### 2.2 Specimen collection

A total of 138 patients’ bronchoalveolar lavage fluid (BALF) samples were collected to analyze the respiratory tract microbiota, with each sample labeled according to patient details. The collection procedure involved several steps as follows: In cases of localized lesions, the segment containing the lesion was chosen. For diffuse lesions, the most severe segment was selected (Pan et al., 2022). The bronchoscope tip was positioned in the target bronchial segment or sub-end opening. Sterile physiological saline at a temperature of 37°C or room temperature was injected rapidly through the operating channel in multiple injections of 20–50 mL each, with a total volume ranging from 60–120 mL. Immediately after saline injection, appropriate negative pressure was applied to aspirate the bronchoalveolar lavage fluid, aiming for a recommended total recovery rate of ≥30%. The collected fluid comprised secretions from approximately 10 mL of bronchial terminals and alveoli. Any potentially contaminated portion at the front end was discarded, and the remaining portion, constituting at least approximately 5 mL, was promptly collected into a test tube. The collected BALF samples were stored at -80°C. All samples were obtained from the area of lung infiltration, with priority given to the site of most severe infiltration in cases of multiple infiltrated areas.

### 2.3 DNA extraction, library preparation, and sequencing

Initially, cell membrane lysis and host DNA depletion were performed on BALF samples. Following this, 250 μl of the post-lysis supernatant was transferred into a 1.5 mL centrifuge tube and combined with 300 μl of a lysis buffer mixture, followed by homogenization through vortexing. After a brief centrifugation, the mixture underwent a 10-minute incubation at 70°C. DNA extraction was performed using a magnetic bead mixture consisting of 350 μl isopropanol and 15 μl magnetic beads. The DNA concentrations were quantified using the Qubit dsDNA HS Assay Kit (Thermo Fisher Scientific). Subsequently, the DNA underwent fragmentation, end-complementation, and sequencing adapter ligation following the library construction protocol. Finally, the libraries were sequenced using the Vision 1000 high-throughput sequencing platform, targeting an output of 20 million 50 bp single-end sequence data per read.

### 2.4 Bioinformatics analysis

Data quality control and species classification: To ensure the accuracy and reliability of subsequent information analysis results, raw sequencing data undergoes filtering and processing using fastp software to obtain quality-controlled data. Bowtie2 aligns the sequences to the host genome, removing host-aligned sequences. Kraken2 annotates and classifies all effective sequences of the samples to study species composition and diversity information. Bracken re-estimates species composition abundance and excludes background contaminating DNA interference, utilizing negative controls in each mNGS run. Top 15 species by abundance are visualized using the R package ggplot2, with statistical testing via the Wilcoxon rank-sum test.

Alpha diversity analysis: Alpha diversity, measuring abundance and diversity of microbial communities, employs statistical indices estimating species richness and diversity for each sample. The Chao index, estimating species count using the Chao1 algorithm, and the Ace index estimate total species richness. The Shannon index assesses microbial diversity, while the Simpson index quantifies biodiversity. Alpha diversity indices are calculated for each sample using the R package Vegan.

Beta diversity analysis: Beta diversity compares microbial community compositions of different samples or groups. Principal Coordinate Analysis (PCoA) extracts significant elements capturing sample differences, visualized on a two-dimensional plot. Bray Curtis distance measures dissimilarity between samples, while UniFrac distance computes PCoA analysis in the R package Vegan. The p-value for PCoA analysis is calculated using adonis, and boxplot p-values using the Wilcoxon rank-sum test.

Differential species analysis: LEfSe (Linear discriminant analysis Effect Size) identifies biomarkers in high-dimensional data. It detects species with significant abundance differences between groups using the Kruskal-Wallis rank-sum test. Subsequently, the Wilcoxon rank-sum test assesses differential species consistency across subgroups. Linear regression analysis (LDA) estimates the influence magnitude of abundance for each component. Differential species between groups are calculated using LEfSe software with thresholds of LDA>=2 and p-value<=0.05.

### 2.5 Statistics analysis

In the clinical data, continuous variables with a normal distribution are presented as mean ± standard deviation (SD), whereas non-normally distributed variables are presented as median (Q1, Q3). Statistical analysis involves the application of Student’s t-test and Wilcoxon rank-sum test. Categorical variables are depicted as percentages and scrutinized using the Chi-square test or Fisher’s exact test. A two-sided p-value < 0.05 is regarded as statistically significant in all instances. Machine learning feature selection and statistical analysis were carried out using R version 4.2.3 and Python version 3.10.9.

## 3 Results

### 3.1 Sample characteristics

**Figure 1:**
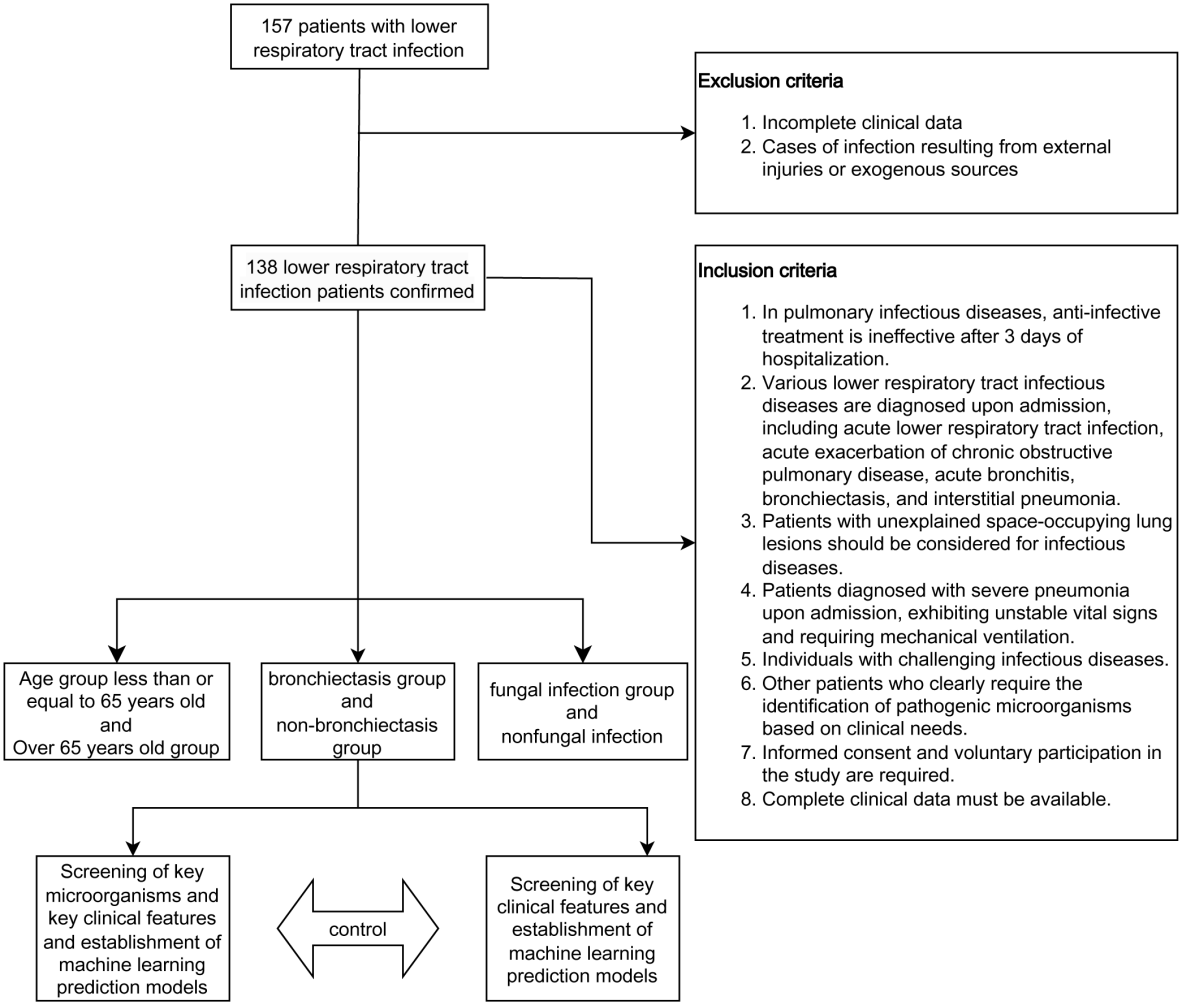
Study design and flow diagram.

The workflow is depicted in **Figure 1**. In this study, a cohort of 138 patients diagnosed with lower respiratory tract infections was recruited. Based on their clinical characteristics, patients were stratified into three subgroups according to age (>65 years or ≤65 years), presence or absence of bronchiectasis, and presence or absence of fungal infections (**Table 1**). Specifically, the cohort comprised 72 patients aged over 65 and 66 patients aged 65 or younger. Among these, 23 patients exhibited bronchiectasis while 115 did not. Furthermore, fungal infections were present in 50 patients, while 88 patients did not exhibit fungal infections. The spectrum of lower respiratory tract infections included community-acquired pneumonia, acute exacerbation of chronic obstructive pulmonary disease, bronchiectasis, obstructive pneumonia, acute bronchitis, and asthma.

**Table 1:** Patients and sample characteristics in three groups, including biochemical parameters and treatment medications.

group	old (n = 72)	young (n = 66)	p	bro (n = 23)	N (n = 115)	p	fun (n = 50)	N (n = 88)	p
age, Median (Q1,Q3)	74.5 (70, 80)	53.5 (43.25, 59)	< 0.001	63 (57.5, 76.5)	66 (52.5, 73)	0.507	70 (59, 78.75)	62.5 (51, 71)	0.003
gender, n (%)			0.203			0.303			0.005
female	22 (31)	28 (42)		11 (48)	39 (34)		10 (20)	40 (45)	
male	50 (69)	38 (58)		12 (52)	76 (66)		40 (80)	48 (55)	
NIPPV, n (%)			0.003			0.771			0.006
N	51 (71)	61 (92)		18 (78)	94 (82)		34 (68)	78 (89)	
Y	21 (29)	5 (8)		5 (22)	21 (18)		16 (32)	10 (11)	
days of hospitalization, Median (Q1,Q3)	16.5 (10, 34)	11 (8, 15.75)	< 0.001	11 (8, 23.5)	13 (9, 23)	0.548	16.5 (12, 29.75)	11 (8, 20)	< 0.001
2hPG, Median (Q1,Q3)	5.27 (4.49, 7.72)	5.03 (4.45, 6.81)	0.417	5.69 (4.64, 9.04)	5.16 (4.45, 7.6)	0.304	5.23 (4.31, 7.58)	5.1 (4.49, 7.61)	0.7
HbA1c, Median (Q1,Q3)	6.4 (5.3, 9.5)	8.3 (5.3, 10.62)	0.925	5.6 (5.3, 9.45)	7 (5.3, 9.5)	0.674	6.4 (5.3, 9.5)	7.15 (5.3, 9.5)	0.955
TG, Median (Q1,Q3)	0.96 (0.65, 1.41)	1 (0.64, 1.49)	0.993	0.83 (0.68, 1.41)	1.01 (0.65, 1.43)	0.793	0.97 (0.63, 1.31)	0.98 (0.7, 1.5)	0.328
total cholesterol, Mean ± SD	3.76 ± 1.23	3.78 ± 0.93	0.875	4.24 ± 1.04	3.67 ± 1.08	0.024	3.54 ± 1.14	3.9 ± 1.04	0.076
HDL-C, Median (Q1,Q3)	1.02 (0.75, 1.42)	0.98 (0.71, 1.21)	0.373	1.02 (0.86, 1.44)	0.97 (0.7, 1.27)	0.346	0.92 (0.68, 1.45)	1 (0.86, 1.26)	0.187
LDL-C, Mean ± SD	2.23 ± 0.8	2.17 ± 0.71	0.654	2.31 ± 0.88	2.18 ± 0.73	0.516	2.07 ± 0.82	2.28 ± 0.71	0.125
TBIL, Median (Q1,Q3)	9.12 (7.3, 11.09)	9.12 (7.3, 11.55)	0.733	9.43 (7.27, 11.01)	8.97 (7.31, 11.58)	0.966	9.55 (7.03, 12.31)	8.91 (7.41, 11.39)	0.789
TP, Median (Q1,Q3)	62.8 (58.08, 65.38)	64.65 (60.75, 70.05)	0.006	62.9 (60.4, 68)	63.69 (60, 67.59)	0.613	61.1 (57.22, 64.89)	64.55 (61.48, 68.65)	0.002
ALB, Mean ± SD	31.5 ± 5.68	35.44 ± 4.94	< 0.001	34.04 ± 4.05	33.25 ± 5.95	0.439	31.3 ± 6.02	34.56 ± 5.14	0.002
globulin, Median (Q1,Q3)	30.3 (26.2, 34.35)	28.95 (26.08, 33.05)	0.477	28.3 (25.3, 33.1)	29.96 (26.5, 34.59)	0.293	28.25 (25.73, 34.2)	30.1 (26.45, 34)	0.494
alanine aminotransferase, Median (Q1,Q3)	20.55 (12.47, 29.35)	26.69 (14.12, 40.85)	0.025	23 (16.45, 37.85)	20.6 (12.65, 34.65)	0.396	24.25 (13.9, 37.83)	20.2 (12.73, 34.35)	0.365
AST, Median (Q1,Q3)	23.45 (17.9, 31.27)	20.55 (17.12, 28.8)	0.24	26.6 (17.2, 30.65)	21.1 (17.4, 28.8)	0.368	24.4 (18.88, 31.37)	20.1 (16.9, 27.55)	0.028
AST-ALT rate, Median (Q1,Q3)	1.23 (0.92, 1.64)	0.96 (0.69, 1.27)	< 0.001	0.98 (0.75, 1.23)	1.1 (0.81, 1.56)	0.239	1.13 (0.74, 1.56)	1.05 (0.8, 1.5)	0.667
LDH, Median (Q1,Q3)	204 (160.43, 257.35)	172.2 (138.95, 221.98)	0.014	181 (157.05, 208.65)	181 (149.97, 240.1)	0.635	217.05 (176.99, 287.79)	168.68 (142.4, 220.4)	< 0.001
WBC, Median (Q1,Q3)	7.7 (6.2, 10)	6.61 (4.94, 8.28)	0.036	7.6 (5.95, 11.52)	6.91 (5.48, 8.88)	0.34	8 (6.77, 13.37)	6.58 (5.22, 8.2)	< 0.001
NEU, Median (Q1,Q3)	5.61 (3.42, 7.84)	4.31 (2.66, 7.15)	0.075	5.08 (3.62, 10.31)	4.7 (3.05, 7.07)	0.243	6.9 (4.52, 12.43)	4.03 (2.79, 5.87)	< 0.001
NEU%, Mean ± SD	73.76 ± 12.94	66.4 ± 14.3	0.002	72.48 ± 14.54	69.79 ± 13.97	0.422	77.16 ± 12.78	66.31 ± 13.26	< 0.001
Lym, Mean ± SD	1.35 ± 0.75	1.61 ± 0.64	0.029	1.49 ± 0.79	1.47 ± 0.7	0.895	1.21 ± 0.72	1.63 ± 0.66	< 0.001
lymphocyte percentage, Mean ± SD	19.43 ± 12.11	25.49 ± 11.77	0.003	19.2 ± 11.96	22.96 ± 12.3	0.181	15.67 ± 8.71	26.11 ± 12.45	< 0.001
PBMC, Median (Q1,Q3)	0.47 (0.33, 0.6)	0.47 (0.33, 0.62)	0.947	0.62 (0.41, 0.94)	0.45 (0.33, 0.56)	0.006	0.54 (0.42, 0.79)	0.42 (0.32, 0.54)	0.006
PBMC%, Mean ± SD	6.28 ± 2.48	6.76 ± 2.07	0.212	6.53 ± 2.48	6.5 ± 2.27	0.957	6.3 ± 2.69	6.63 ± 2.05	0.465
RBC, Median (Q1,Q3)	3.72 (3.43, 4.26)	4.24 (3.92, 4.59)	< 0.001	3.91 (3.66, 4.23)	4.07 (3.55, 4.47)	0.619	3.84 (3.49, 4.3)	4.14 (3.7, 4.47)	0.076
PLT, Median (Q1,Q3)	210.5 (164, 267.75)	246 (195.5, 296)	0.028	247 (216.5, 293.5)	221 (171, 286.5)	0.131	222 (163.5, 264.75)	226.5 (192.5, 291)	0.353
ESR, Median (Q1,Q3)	71 (51, 100.5)	63.5 (32.5, 93)	0.198	66 (26.5, 79)	70 (41, 104.5)	0.076	68 (41, 105.75)	66 (36.75, 97.25)	0.521
C-reactive protein, Median (Q1,Q3)	13.39 (2.51, 47.07)	3.86 (0.8, 25.98)	0.026	5.63 (0.85, 43.41)	8.82 (1.25, 32.7)	0.913	24.04 (5.04, 48.66)	4.4 (0.8, 18.86)	< 0.001
procalcitonin, Median (Q1,Q3)	0.1 (0.04, 0.27)	0.06 (0.04, 0.21)	0.344	0.17 (0.04, 0.45)	0.07 (0.04, 0.2)	0.117	0.1 (0.05, 0.42)	0.07 (0.03, 0.2)	0.009
interleukin-6, Median (Q1,Q3)	3.63 (0.02, 32.33)	3.19 (0.01, 23.05)	0.209	0.05 (0.01, 24)	3.27 (0.01, 31.8)	0.284	3.63 (0.12, 34.1)	3.27 (0.01, 24)	0.11
Ccr, Median (Q1,Q3)	71.55 (53.2, 85.52)	95.5 (74.42, 110.72)	< 0.001	83.5 (62.6, 98.25)	77.5 (67, 98.8)	0.938	75.6 (65, 89.05)	86.8 (67, 106.2)	0.078
Scr, Median (Q1,Q3)	65.2 (54, 83.3)	62.6 (55.75, 74.95)	0.457	62.2 (52.25, 70.2)	65.2 (54.8, 77.68)	0.384	63.65 (56.2, 80.49)	65.47 (53.83, 76.59)	0.723
β2 microglobulin, Median (Q1,Q3)	2.65 (1.73, 3.31)	1.89 (1.53, 2.55)	< 0.001	1.76 (1.29, 3)	2.21 (1.73, 2.98)	0.108	2.37 (1.84, 3.02)	2.06 (1.54, 2.9)	0.153
Potassium, Mean ± SD	3.91 ± 0.55	3.82 ± 0.45	0.268	3.91 ± 0.47	3.86 ± 0.51	0.652	3.83 ± 0.54	3.89 ± 0.49	0.571
sodium, Mean ± SD	140.25 ± 4.56	139.67 ± 2.89	0.369	140.35 ± 3.81	139.9 ± 3.87	0.61	140.67 ± 4.19	139.58 ± 3.61	0.126
Chlorine, Median (Q1,Q3)	104.9 (102.15, 108.15)	106.1 (103.81, 108.27)	0.1	105.4 (102.02, 107.4)	105.8 (103.02, 108.3)	0.355	103.99 (101.23, 107.55)	106.05 (103.88, 108.3)	0.012
calcium, Mean ± SD	2.1 ± 0.13	2.19 ± 0.13	< 0.001	2.12 ± 0.12	2.15 ± 0.14	0.343	2.1 ± 0.15	2.17 ± 0.13	0.014
Inorganic phosphate, Mean ± SD	0.95 ± 0.24	1.1 ± 0.27	< 0.001	1.05 ± 0.35	1.01 ± 0.24	0.684	0.94 ± 0.29	1.06 ± 0.24	0.016
magnesium, Median (Q1,Q3)	0.86 (0.79, 0.94)	0.86 (0.79, 0.91)	0.604	0.88 (0.82, 0.93)	0.86 (0.79, 0.92)	0.266	0.83 (0.78, 0.93)	0.86 (0.8, 0.92)	0.359
uric acid, Median (Q1,Q3)	245.65 (180.34, 306.28)	245.6 (189.4, 314.17)	0.664	227.3 (174.8, 297.9)	251.4 (188.3, 307.55)	0.704	218.15 (155.27, 261.52)	268.27 (198.38, 320.4)	0.002
iron, Median (Q1,Q3)	8.38 (4.78, 12.91)	10.88 (6.62, 14.64)	0.081	10.17 (6.41, 13.06)	9.2 (5.76, 14.27)	0.97	6.75 (4.19, 10.7)	11.86 (7.33, 14.99)	< 0.001

NIPPV, non invasive positive pressure ventilation; 2hPG, 2-hour postprandial blood glucose; HbA1c, Hemoglobin A1C; TG, Triglycerides; TC, total cholesterol; HDL-C, high density lipoprotein cholesterol; LDL-C, low density lipoprotein cholesterolcholesterol; TBIL, total bilirubin; TP, Total protein; ALB, Albumin; GLB, globulin; ALT, Alanyl aminotransferase; AST, aspartate aminotransferase; LDH, lactate dehydrogenase; WBC, White blood cell count; NEU, neutrophil count;NEU%, Neutrophil percentage; Lym, lymphocyte count; Lym%, lymphocyte percentage; PBMC, Peripheral blood mononuclear cell; RBC, red blood cell count; PLT, platelet count; ESR, Erythrocyte sedimentation rate measurement; PCT, procalcitonin;CRP, C-reactive protein; IL-6, interleukin-6; Ccr, endogenous creatinine clearance; Scr, Serum creatinine; β2-MG, β2 microglobulin; UA, uric acid.

### 3.2 Distinct bacterial types and intergroup variations exist among different subgroups

#### 3.2.1 Age groups

**Figure 2:**
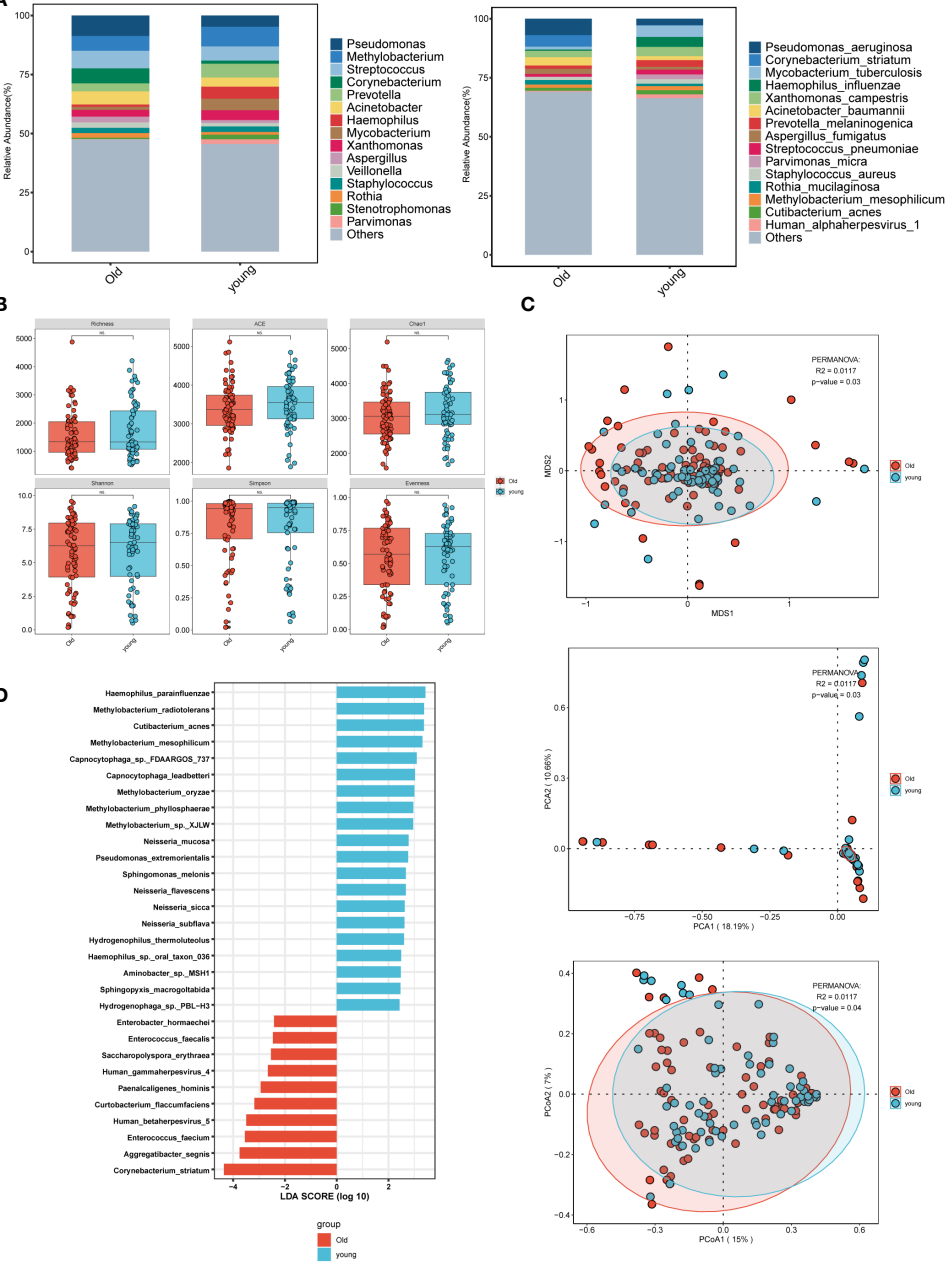
Microbial characteristics across age groups in patients with pulmonary infections. **(A)** The distribution of the top 15 microbial taxa at the genus level and species level. **(B)** Analysis of microbial alpha diversity. **(C)** Analysis of microbial beta diversity. **(D)** LEfSe analysis was performed on young and old groups of microorganisms to demonstrate the distribution of the top 30 microorganisms at the species level.

In the age group comparison (**Figure 2A**), the top 15 microorganisms at the genus level in the elderly group were ranked by their relative abundance. These microorganisms included *Pseudomonas*, *Streptococcus*, *Corynebacterium*, *Methylobacterium*, *Acinetobacter*, *Prevotella*, *Xanthomonas*, *Aspergillus*. At the species level, prevalent species were identified as *Pseudomonas aeruginosa*, *Corynebacterium striatum*, *Acinetobacter baumannii*, *Aspergillus fumigatus*, *Human alphaherpesvirus 1*, and *Parvimonas micra*. In the younger group, the predominant 15 microorganisms, including bacteria, fungi, and viruses, consisted of *Methylobacterium*, *Streptococcus*, *Prevotella*, *Haemophilus*, and *Mycobacterium* at the genus level, and *Mycobacterium tuberculosis*, *Haemophilus influenzae*, and *Xanthomonas campestris* at the species level. *Pseudomonas* was most abundant at the genus level in the elderly group, while *Methylobacterium* was highest in the younger group. At the species level, *Mycobacterium tuberculosis* was highest in the younger group. Although there was no statistically significant difference in α-diversity between the younger and elderly groups (**Figure 2B**), β-diversity analysis, including PCA, PCoA, and NMDS (**Figure 2C**), revealed significant differences, indicating meaningful grouping between the two groups. LEfSe analysis (**Figure 2D**) showed more common pathogenic microbial species in the elderly group, such as *Human gammaherpesvirus 4* (*EBV*), *Human betaherpesvirus 5*, and *Enterococcus faecalis*, while the majority of the younger group consisted of oral and upper respiratory symbiotic bacteria.

#### 3.2.2 Merging the bronchiectasis groups

**Figure 3:**
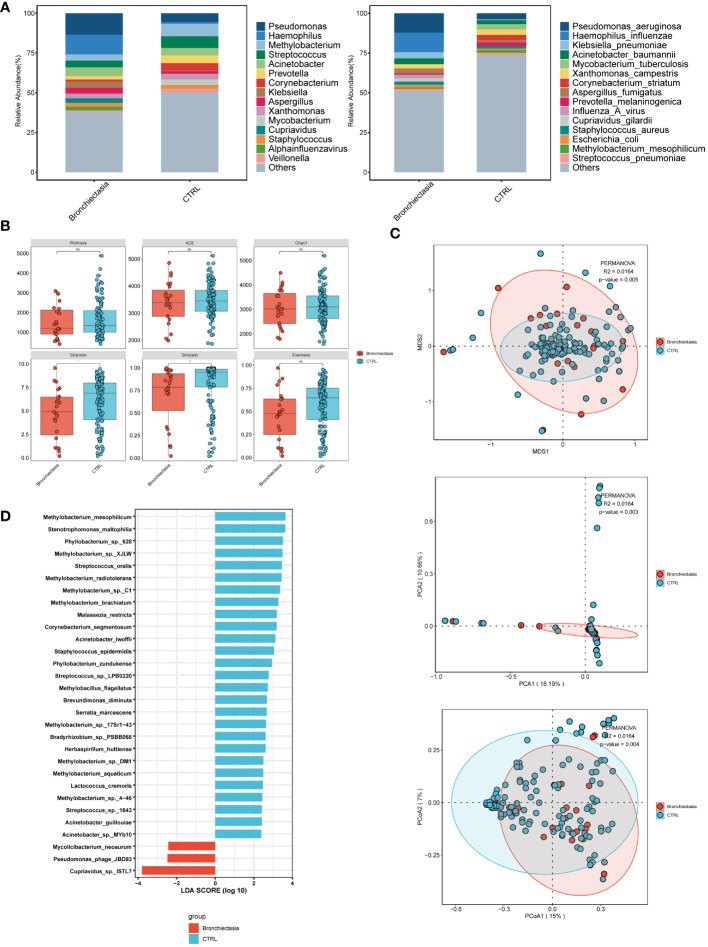
Microbial characteristics in patients with merging bronchiectasis and non-merging group in lower respiratory tract infections. **(A)** The distribution of the top 15 microbial taxa at the genus level and species level. **(B)** Analysis of microbial alpha diversity. **(C)** Analysis of microbial beta diversity. **(D)** LEfSe analysis was performed on two groups of microorganisms to demonstrate the distribution of the top 30 microorganisms at the species level.

In the comparison between patients with and without bronchiectasis (**Figure 3A**), the bronchiectasis group exhibited a higher abundance of *Pseudomonas*, *Haemophilus*, *Acinetobacter*, *Streptococcus*, *Klebsiella*, *Aspergillus*, and *Alpha influenzavirus* among the top 15 microorganisms at the genus level, ranked by relative abundance. At the species level, dominant species included *Pseudomonas aeruginosa*, *Haemophilus influenzae*, *Klebsiella pneumoniae*, *Acinetobacter baumannii*, *Aspergillus fumigatus*, and *Influenza A virus*. Conversely, in the non-bronchiectasis group, the top 15 microorganisms at the genus level were *Methylobacterium*, *Streptococcus*, *Pseudomonas*, *Prevotella*, *Acinetobacter*, *Aspergillus*, and *Alpha influenzavirus*. At the species level, prevailing species were *Pseudomonas aeruginosa*, *Mycobacterium tuberculosis*, *Xanthomonas campestris*, *Corynebacterium striatum*, *Acinetobacter baumannii*, *Aspergillus fumigatus*, and *Influenza A virus*. Notably, the bronchiectasis group exhibited higher abundance of *Pseudomonas* and *Pseudomonas aeruginosa* at both the genus and species levels, followed by *Haemophilus (Haemophilus influenzae)* infection. The non-bronchiectasis group showed a higher abundance of *Methylobacterium* and *Streptococcus* at the genus level, and *Pseudomonas aeruginosa* and *Mycobacterium tuberculosis* at the species level. Both groups had a similar prevalence of fungal and viral infections. Analysis of α-diversity (**Figure 3B**) revealed that the bronchiectasis group had lower Shannon and Simpson indices compared to the non-bronchiectasis group, indicating decreased microbial diversity. Furthermore, the analysis of β-diversity (**Figure 3C**) including PCA, PCoA, and NMDS showed statistically significant differences (p<0.05) between the two groups, highlighting distinct microbial compositions. Results from LEfSe analysis (**Figure 3D**) indicated significant differences in bacterial composition, with an enrichment of *Cupriavidus* sp. *ISTL7*, *Pseudomonas phage JBD93*, and *Mycolicibacterium neoaurum* in the bronchiectasis group. In contrast, the non-bronchiectasis group predominantly consisted of normal skin and oral flora, including *Methylobacterium*, *Streptococcus oralis*, and *Phyllobacterium* sp. *628*.

#### 3.2.3 Merging the fungal infection groups

**Figure 4:**
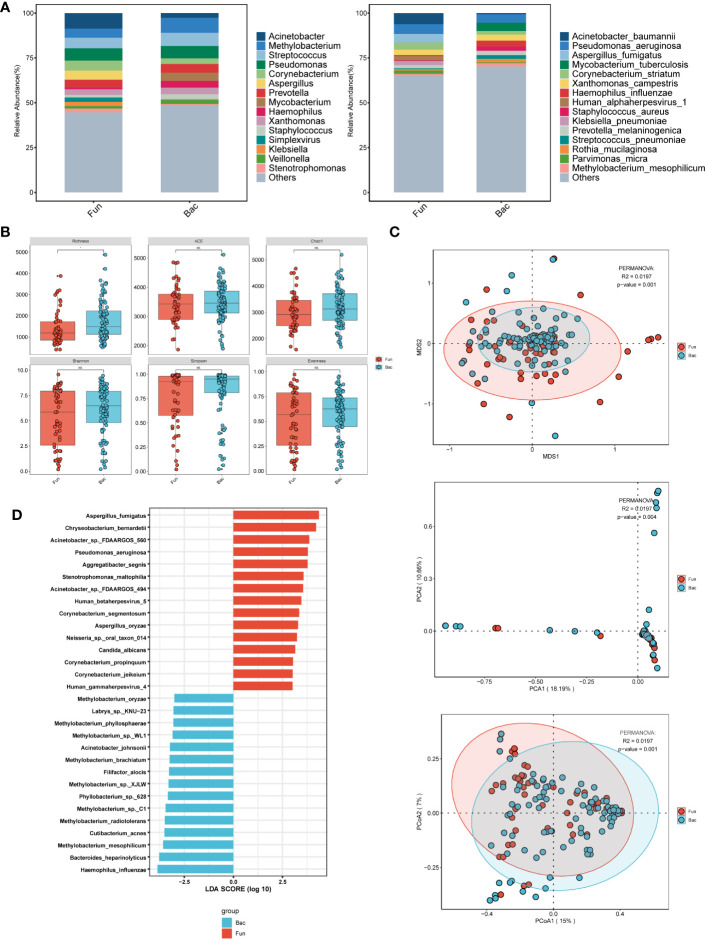
Microbial characteristics in patients with merging fungal infections and non-merging group in lower respiratory tract infections. **(A)** The distribution of the top 15 microbial taxa at the genus level and species level. **(B)** Analysis of microbial alpha diversity. **(C)** Analysis of microbial beta diversity. **(D)** LEfSe analysis was performed on two groups of microorganisms to demonstrate the distribution of the top 30 microorganisms at the species level.

In the grouping of fungal and bacterial infections (**Figure 4A**), we observed that the fungal infection group exhibited a higher abundance of *Acinetobacter*, *Pseudomonas*, *Klebsiella*, and *Stenotrophomonas* at the genus level. Aspergillus predominated among fungal infections, while viral infections included Simplexvirus. At the species level, dominant microorganisms in the fungal infection group included *Acinetobacter baumannii, Pseudomonas aeruginosa, Aspergillus fumigatus*, and *Corynebacterium striatum*, while viral infections featured *Human alphaherpesvirus 1*. In contrast, the group without fungal infections showed a higher abundance of *Methylobacterium* at the genus level, and *Pseudomonas aeruginosa* and *Mycobacterium tuberculosis* at the species level. α-diversity analysis (**Figure 4B**) indicated that the non-fungal infection group had higher Richness compared to the fungal infection group, suggesting increased abundance of microbial taxa and a more diverse and stable microbial ecosystem. β-diversity analysis (**Figure 4C**) also revealed significant differences in microbial composition between the two groups, indicating meaningful grouping. Moreover, LEfSe analysis (**Figure 4D**) demonstrated distinct microbial compositions in the fungal infection group compared to the non-fungal infection group, with notable bacteria such as *Pseudomonas aeruginosa*, *Acinetobacter*, and *Chryseobacterium bernardetii*, and viruses including *Human gammaherpesvirus 4* (*EBV*) and *Human betaherpesvirus 5*. Additionally, there were differential abundances of fungal species such as *Candida albicans*, *Aspergillus oryzae*, and *Aspergillus fumigatus* in the fungal infection group. In contrast, the non-fungal infection group was characterized by higher abundances of *Methylobacterium* at the genus level, and *Haemophilus influenzae*, *Cutibacterium acnes*, *Filifactor alocis*, and *Labrys* sp. *KNU 23* at the species level.

### 3.3 Correlation analyses

**Figure 5:**
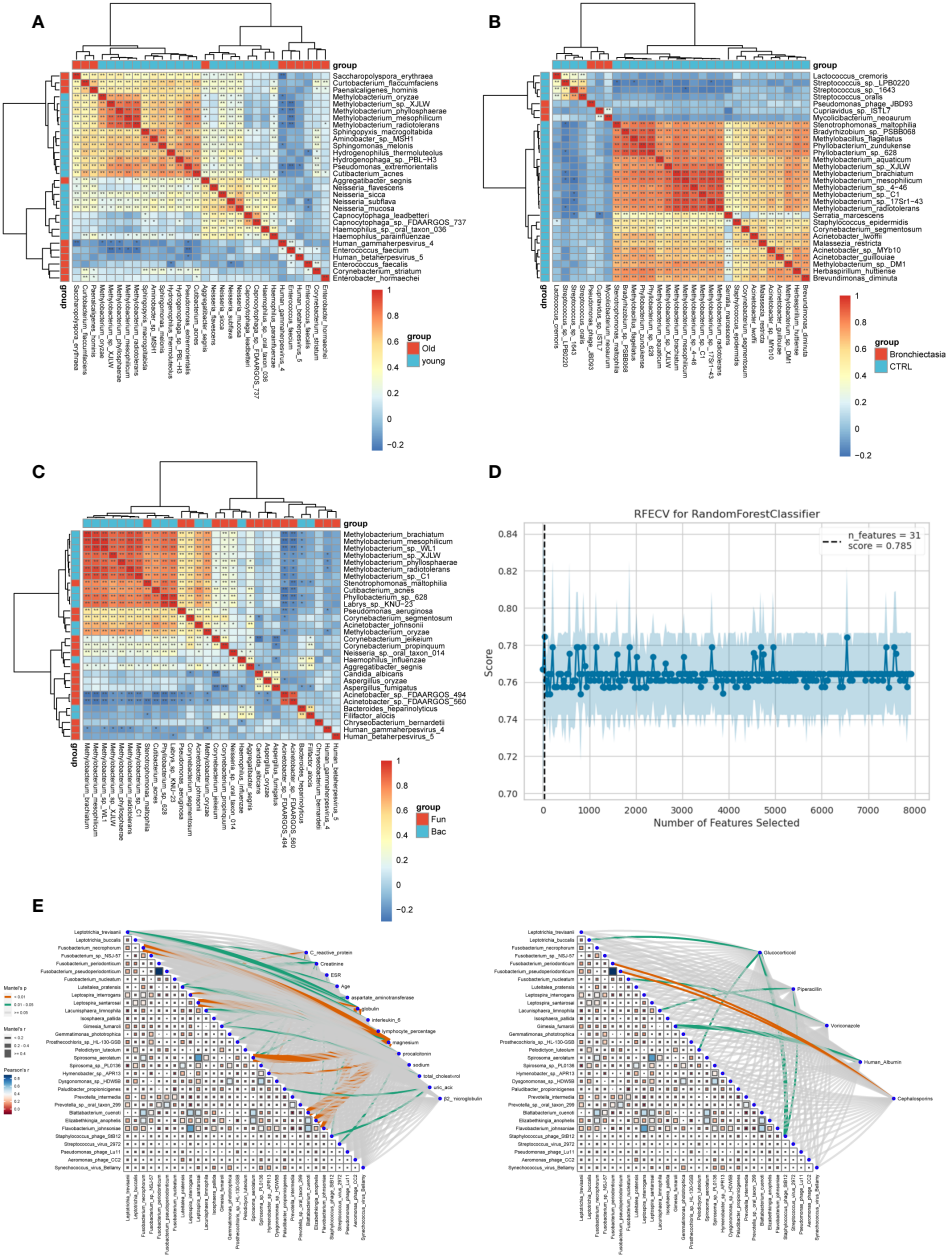
Correlation analysis was performed between two groups within each cluster and the key bacteria in the bronchiectasis group. **(A–C)** Microbial correlations among the control groups of different age groups, bronchiectasis groups, and fungal groups were analyzed based on the top 30 microorganisms identified through LEfSe analysis. **(D)** A total of 31 bacterial features were selected based on RFECV feature selection from the bronchiectasis group and the control group. **(E)** Based on the clinical data characteristics of the patients, feature variables were selected through machine learning. After screening, 31 key bacteria, 14 clinical indicators, and 14 medications were identified in the bronchiectasis group. Correlation analysis was then conducted. Left panel: Analysis of the correlation between clinical indicators and key bacteria. Right panel: Analysis of the correlation between medications and key bacteria on the right.

Next, correlation analysis was conducted on the top 30 bacteria in the six groups based on an LDA threshold of LDA>=2 and p-value <= 0.05. Positive correlations were observed between *Methylobacterium*, *Neisseria*, and *Capnocytophaga* at the species level in the young group, while a negative correlation was noted between *Methylobacterium* and *Human gammaherpesvirus 4 (EBV)*, *Enterococcus faecium* in the elderly group (**Figure 5A**). Furthermore, the analysis (**Figure 5B**) revealed positive correlations between *ycolicibacterium neoaurum* and *Cupriavidus sp ISTL7* in the bronchiectasis group with merging bronchiectasis, while the non-merging bronchiectasis group showed positive correlations among most symbiotic bacteria. Subsequently, significant negative correlations were identified between *Acinetobacter* bacteria (*Acinetobacter sp FDAARGOS 494*, *Acinetobacter sp FDAARGOS 560*) and certain bacterial species from *Methylobacterium, Stenotrophomonas maltophilia, Cutibacterium acnes, Phyllobacterium sp 628, Labrys sp KNU 23*, and *Pseudomonas aeruginosa* in the merging fungal infection group (**Figure 5C**).

To explore the association between clinical medication, clinical indicators, and bacterial presence in clinical diagnosis and treatment, a machine learning approach was employed to identify key variables for the bronchiectasis group based on patients’ clinical characteristics. A total of 31 key bacteria (**Figure 5D**), 14 clinical indicators, and 14 medications were identified through feature selection. Subsequently, correlation analysis was performed. Significant associations were observed between *Luteitalea pratensis* and key markers of inflammation, including C-reactive protein, Procalcitonin, and lymphocyte percentage. *Hymenobacter* sp. *APR13* exhibited correlations with C-reactive protein and Procalcitonin, while *Staphylococcus phage StB12* was associated with erythrocyte sedimentation rate (ESR). *Streptococcus virus 2972* showed a correlation with Procalcitonin, and Leptotrichia buccalis demonstrated a correlation with interleukin-6. In terms of liver function markers, Gimesia fumaroli displayed correlations with aspartate aminotransferase and total cholesterol. Prosthecochloris sp. HL-130-GSB and *Spirosoma aerolatum* were related to *aspartate aminotransferase*. Additionally, correlations were observed among *Elizabethkingia anophelis, Fusobacterium necrophorum, and magnesium, globulin*, and *creatinine*. In the correlation between bacteria and drugs (**Figure 5E** right), associations were noted between *Glucocorticoids* and *Leptotrichia buccalis, Pelodictyon luteolum*, and *Staphylococcus* phage StB12. *Piperacillin* was associated with *Lacunisphaera limnophila* and *Staphylococcus* phage StB12, while Voriconazole was correlated with Fusobacterium nucleatum. Human albumin injection showed relationships with *Gimesia fumaroli*, and cephalosporin drugs demonstrated associations with *Fusobacterium pseudoperiodonticum* and *Fusobacterium periodonticum*.

### 3.4 Machine learning prediction models

**Figure 6:**
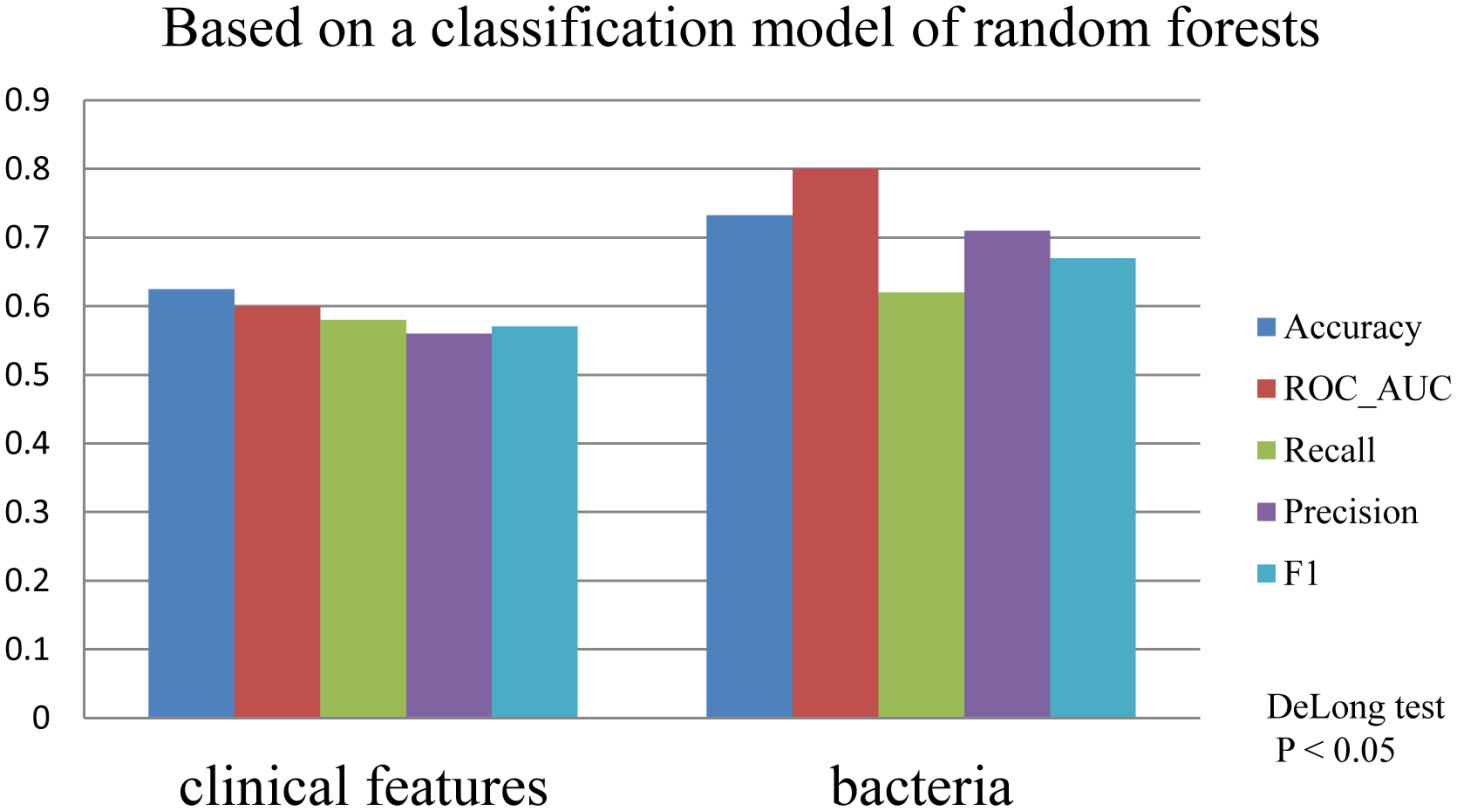
** **A comparison of machine learning models under different predictor variables was conducted. Based on microbial and clinical indicators, machine learning models using random forest were constructed to predict patients’ length of hospital stay. The term “Accuracy” refers to the proportion of correctly classified samples to the total number of samples. The Receiver Operating Characteristic (ROC) curve is a comprehensive indicator that reflects the sensitivity and specificity of continuous variables. “Precision,” also known as positive predictive value, represents the proportion of true positive samples among predicted positive samples. “Recall,” also known as sensitivity, represents the proportion of true positive samples among all positive samples. The F1 score is a weighted average of precision and recall. Delong test, p<0.05(0.0284629), the statistical significance was significant.

To evaluate the predictive influence of bacteria on hospitalization duration in patients with lower respiratory tract infections, we divided the hospital stay days of all patients into two groups: a short group and a long group, based on the median value of 13. Furthermore, we utilized machine learning techniques to develop a predictive model, incorporating the selected variables (**Supplementary Tables S4, S5**). Remarkably, in the random forest models constructed separately based on clinical indicators and bacteria, the model incorporating bacteria demonstrated superior predictive performance (**Figure 6**).

## 4 Discussion

### 4.1 Age

*Pseudomonas aeruginosa* is a Gram-negative bacterium that can survive in various environments and is widely distributed (Silby et al., 2011). As the age of patients infected with *Pseudomonas aeruginosa* increases, the number of drug-resistant strains also increases (Hu et al., 2019). In the elderly group, the most prevalent genera in BALF are *Pseudomonas*, with *Pseudomonas aeruginosa* being the dominant species. In contrast, the microbial abundance in the young group is noticeably different. *Mycobacterium tuberculosis*, the causative agent of *tuberculosis*, shows a significant correlation with age, with a higher likelihood of transmission and infection in younger individuals (Borgdorff et al., 2001; Yang et al., 2012). *Haemophilus influenzae*, a common inhabitant of the oral and respiratory tracts, is a characteristic respiratory microbiota dominated by *Streptococcus* and *Haemophilus* (*Haemophilus influenzae*) in the young group compared to the old group. Additionally, *Corynebacterium striatum*, a highly abundant species in the genus *Corynebacterium*, is normally found in various mucosal locations such as human skin and the nasopharynx (Funke et al., 1997). There are reports suggesting that *Corynebacterium striatum* is increasingly recognized as an infection-related bacterium (Díez-Aguilar et al., 2013; Yang et al., 2018). In 2018, researchers in the United States reported three cases of community-acquired pneumonia (CAP) with strains predominantly belonging to the genus *Corynebacterium*, indirectly indicating a close association between the genus *Corynebacterium* and lower respiratory tract infections (Yang et al., 2018). However, most elderly individuals have compromised immune function, which is a prerequisite for *Corynebacterium* infection (Nudel et al., 2018; Lee et al., 2022). *Acinetobacter baumannii* is the most common bacterium in mechanically ventilated patients (Xie et al., 2018), and the rising antimicrobial resistance of *Acinetobacter* has led to a broader recognition that it is no longer exclusively a nosocomial pathogen in elderly individuals. It is extensively prevalent in long-term acute care facilities, nursing homes, and the community (Sengstock et al., 2010). Our results show that the majority of patients infected with *Acinetobacter baumannii* are in the old group. In the old group, *Methylobacterium mesophilicum* and *Rothia mucilaginosa* are opportunistic infection bacteria associated with immunodeficiency (Sanders et al., 2000; Chavan et al., 2013; Maraki and Papadakis, 2015), although only a few cases have been reported (Sanders et al., 2000; Engler and Norton, 2001; Maraki and Papadakis, 2015). While individual cases of *Rothia mucilaginosa* infection have been reported in patients with normal immune function (Baeza Martínez et al., 2014), it is not a primary consideration for lower respiratory tract infections in patients. As for *Prevotella*, it primarily resides in the intestines and oral cavity (Tett et al., 2021). Although the diversity of *Prevotella* is related to the host’s diet and lifestyle, it also plays a critical role in maintaining human health and disease (Tett et al., 2021). Related studies have shown that *Prevotella* can regulate inflammatory responses (Marietta et al., 2016) and some lung inflammation (Bernasconi et al., 2016), may be associated with respiratory dysbiosis (Welp and Bomberger, 2020). In terms of fungi, we also observed *Aspergillus fumigatus*, a representative of the *Aspergillus* genus. *Aspergillus fumigatus* is the most common species in the *Aspergillus* genus, but fungal infections often have few notable characteristics and the pathogen may not be detectable for a long time. Therefore, diagnosing fungal infections in patients with normal immune function can be challenging, especially as age increases and immune function declines (Zhang et al., 2014; Zhou et al., 2023). A multicenter retrospective study has shown that virus reactivation is associated with an increased risk of mortality in patients (Huang et al., 2023). Our LEfSe analysis reveals that the old group has a greater number of common pathogenic microorganism species compared to the young group, such as *Human gammaherpesvirus 4* (*EBV*) and *Human betaherpesvirus 5*. In the young group, the majority of microorganisms are symbiotic bacteria in the oral cavity and upper respiratory tract, whereas a negative correlation exists between *Methylobacterium* and *Human gammaherpesvirus 4* (*EBV*) and *Enterococcus faecium* in the old group.

### 4.2 Bronchiectasis

*Pseudomonas aeruginosa* is among the most commonly isolated pathogens in the sputum of bronchiectasis patients, whether in the stable or exacerbation phase of the disease in clinical settings (Tunney et al., 2013; Lin et al., 2016). Moreover, *Pseudomonas aeruginosa* is a significant risk factor for the severity and prognosis of bronchiectasis (Loebinger et al., 2009; Wang et al., 2018). In the combined bronchiectasis group, *Pseudomonas* is the most abundant genus, with *Pseudomonas aeruginosa* being the predominant species. In the non-merging bronchiectasis group, *Methylobacterium* genus belongs to the opportunistic infection bacteria (Sanders et al., 2000) and is commonly colonized in various parts of the human body (Kaye et al., 1992; Liu et al., 1997). Additionally, the relative abundance of *Pseudomonas* and *Pseudomonas aeruginosa* is lower than that in merging the bronchiectasis group. Haemophilus influenzae is significantly associated with the severity of bronchiectasis (Purcell et al., 2014). Within the combined bronchiectasis group, we also found that its relative abundance ranks second. In merging bronchiectasis group, their relative abundance ranks second. Additionally, *Klebsiella pneumoniae* is a bacterium that distinguishes the merging bronchiectasis group from non-merging group and aligns with previous studies (Huang et al., 2020). Moreover, our study identified significant differences in *Acinetobacter baumannii*, a bacterium of the *Acinetobacter* genus, between the merging and non-merging bronchiectasis groups. This disparity extends beyond relative abundance to include variations in the presence of other bacteria within the *Acinetobacter* genus across these groups. In terms of fungi and viruses, although there is no significant difference at the species level between the two groups, The relative abundance of common fungi (such as *Aspergillus*) and *Alphainfluenzavirus* was higher in the merging bronchiectasis group compared to the non-merging group. In terms of α-diversity, Many studies have indicated that the occurrence of diseases can lead to a reduction in microbial diversity (Oriano et al., 2020; Liu et al., 2023; Wu et al., 2023), our research similarly demonstrates a decrease in microbial diversity in the merging bronchiectasis group. Particularly noteworthy in the LEfSe analysis is the significant difference in bacteria, including *Cupriavidus* sp. *ISTL7*, which is commonly found in the human environment (Gupta et al., 2019, 2021) and the significant difference in *Pseudomonas phage JBD93*, indirectly reflecting the changes in *Pseudomonas aeruginosa* in this group. This is similar to an arms race between bacteria and phages, and phages may directly participate in interactions with immune cells and play a role in immune regulation (Lepage et al., 2008; Letarov and Kulikov, 2009). The rise of bacterial drug resistance has sparked considerable interest in the relationship between bacteriophages and Pseudomonas aeruginosa (Haddock et al., 2023), along with a renewed focus on phage therapy (Fujiki et al., 2023). *Mycolicibacterium neoaurum*, a *mycobacterium* opportunistic infection, was previously mainly found in immunocompromised individuals (Pang et al., 2022). However, there have been more reports of infections in cases that have undergone invasive medical examinations or surgeries (Shapiro et al., 2023). In the mouse experiment, it was demonstrated that *Mycolicibacterium neoaurum* enhances the suppressive activity of regulatory T cells (Tregs) and increases the mortality rate in cases of Salmonella co-infection (Wang et al., 2020). We also found a positive correlation between *Mycolicibacterium neoaurum* and *Cupriavidus* sp. *ISTL7* In the merging bronchiectasis group.

### 4.3 Fungus

In the merging fungal infection group, in addition to *Aspergillus*, especially *Aspergillus fumigatus*, which has a relatively high abundance, *Acinetobacter* and *Pseudomonas*, including *Acinetobacter baumannii* and *Pseudomonas aeruginosa*, show the highest abundance. This is consistent with previous studies (Zhao et al., 2021). It has been reported that *Acinetobacter* and fungal infections are correlated as two related pathogenic microorganisms (Thoma et al., 2022). Previous studies have also reported that the colonization of *Candida* in the respiratory tract of patients increases the risk of *Pseudomonas* ventilator-associated pneumonia (Azoulay et al., 2006). In the LEfSe analysis of this group, *Candida albicans*, *Aspergillus oryzae*, and *Aspergillus fumigatus* were found to be significantly different from the non-merging fungal infection group. Therefore, we speculate that there is a strong correlation between *Acinetobacter*, *Pseudomonas aeruginosa*, and fungal infections. Generally, in healthy individuals, innate immunity serves as a barrier against Aspergillus infection. However, individuals with compromised immune function are more vulnerable to Aspergillus infection, particularly in combination with viral infections (Huang et al., 2023). At the viral species level, *Human gammaherpesvirus 4* (*EBV*) and *Human betaherpesvirus 5* are distinguishable from the non-merging fungal infection group. And *Methylobacterium* is the most abundant in the non-fungal infection group. Meanwhile, the non-merging fungal infection group has a higher richness compared to the merging fungal infection group. A higher abundance microbial ecosystem is usually considered more diverse and stable. Additionally, there are species differences between the two groups. The correlation heatmap demonstrates a significant negative correlation between *Acinetobacter* bacteria (*Acinetobacter* sp. *FDAARGOS 494*, *Acinetobacter* sp. *FDAARGOS 560*), and some common environmental bacteria, suggesting possible competition between *Acinetobacter* and these bacteria (Littman and Pamer, 2011).

### 4.4 Correlation analysis

We conducted further analysis on the correlation between drugs and bronchiectasis -associated infection bacteria. Lep*totrichia buccalis* is a normal oral bacterium, and there have been isolated reports of it causing severe infectious cavitary pneumonia and sepsis in immunocompromised patients (Morgenstein et al., 1980). The genus *Staphylococcus* is a common bacterium in the human living environment, and *Staphylococcus phage StB12* is closely associated with *Staphylococcus*. It participates in the encoding and evolution of virulence genes and antibiotic resistance in *Staphylococcus* (Brüssow et al., 2004). Therefore, our study elucidates the correlation between the use of glucocorticoids and penicillin-like drugs, such as pipracillin, and the prevalence of these phages. While phage therapy has a long-standing history and has been extensively explored in medicine (Samson et al., 2023), it remains uncertain whether these drugs are indirectly or directly related to bacteriophages. As for cephalosporin drugs and voriconazole, *Fusobacterium* is the main related bacteria. Literature reports have shown that culturing of *Fusobacterium nucleatum* supernatant induces the expression of SARS-CoV-2 receptor ACE2 and the production of interleukins IL-6 and IL-8 in alveolar epithelial cells, exacerbating SARS-CoV-2 infection (Takahashi et al., 2021). There is also evidence to suggest that *Fusobacterium* may have a potential role in protecting the oral mucosa from SARS-CoV-2 infection (Nardelli et al., 2021). In our study, we demonstrated an association between pipracillin, voriconazole, and the presence of *Fusobacterium*, which requires further experimental research to determine whether *Fusobacterium* is an enemy or friend in patients with bronchodilator. In the correlation analysis of clinical indicators and infection bacteria related to bronchiectasis. Previous studies have found a correlation between CRP and Pneumocystis jirovecii in patients with non-HIV immunodeficiency (Zhao et al., 2022), as well as a correlation between monocyte count and fungal infections (Wang et al., 2022). Magnesium is usually considered to have anti-inflammatory effects (Tam et al., 2003), but there are reports that in animal experiments, magnesium may inhibit neutrophil oxidative burst, which is harmful for chronic diseases (Bussière et al., 2002). However, only bacteria such as *Pseudomonas aeruginosa* were discussed, and we speculate that some common environmental bacteria, including *Fusobacterium* and *Prevotella*, are also involved.

### 4.5 Machine learning

Machine learning applications in disease diagnosis (Lai et al., 2024), complication prediction (Pax et al., 2024), and forecasting of factors such as bacterial drug resistance and predictive models for bacteriophage therapy of Escherichia coli urinary tract infections have demonstrated promising predictive efficacy (Hu et al., 2023; Dixit et al., 2024; Keith et al., 2024; Nsubuga et al., 2024). Additionally, numerous clinical machine learning prediction models have been developed to predict disease prognosis and survival time by collecting large-scale clinical features (Kogan et al., 2022; Li et al., 2022; Tang et al., 2022; Li et al., 2023), demonstrating excellent predictive performance. However, in the actual treatment of patients with lower respiratory tract infections (Sethi, 2010; Jain et al., 2015), the complex relationships between microorganisms must be considered. Microorganisms are important reference factors, and it is crucial to understand the relationship between microorganisms and time of hospital stay during the diagnosis and treatment process. The relationship between bacteria and time of hospital stay remains understudied. Our developed machine learning prediction model revealed that incorporating specific bacteria as predictors for the time of hospitalization in cases of lower respiratory tract infections resulted in significantly improved predictive accuracy. This novel insight offers a fresh perspective in patient care, and we anticipate that by advancing our ability to precisely detect microorganisms, we can further tailor individualized treatment strategies.

## 5 Conclusion

In conclusion, we initially scrutinized the microbial community characteristics based on age, the presence of bronchiectasis-related infection, and fungal infection. Furthermore, we examined the correlation between the microbial community and clinical indicators, as well as treatment medications in the bronchodilator group. Finally, leveraging machine learning techniques, we juxtaposed specific microbial features with clinical attributes to assess the predictive efficacy of patient hospital stay duration. These findings elucidate the variances in microbial community characteristics in lower respiratory tract infections across diverse conditions and underscore the potential of bacterial features in forecasting the length of patient hospitalization.

## Data availability statement

The raw sequence data reported in this paper have been deposited in the Genome Sequence Archive (Genomics, Proteomics & Bioinformatics 2021) in National Genomics Data Center (Nucleic Acids Res 2022), China National Center for Bioinformation/Beijing Institute of Genomics, Chinese Academy of Sciences (GSA-Human: HRA007371) that are publicly accessible at https://ngdc.cncb.ac.cn/gsa-human.

## Ethics statement

The studies involving humans were approved by Ethics Committee of the Third People’s Hospital of Chengdu. The studies were conducted in accordance with the local legislation and institutional requirements. The participants provided their written informed consent to participate in this study. Written informed consent was obtained from the minor(s)’ legal guardian/next of kin for the publication of any potentially identifiable images or data included in this article.

## Author contributions

JL: Data curation, Formal analysis, Writing – original draft. AX: Methodology, Formal analysis, Data curation, Writing – review & editing. JW: Data curation, Investigation, Writing – review & editing. XW: Investigation, Writing – review & editing. LB: Investigation, Writing – review & editing. LZ: Data curation, Writing – review & editing. XH: Writing – original draft, Writing – review & editing, Formal analysis, Supervision. GL: Conceptualization, Funding acquisition, Supervision, Writing – review & editing.
